# Middle ear microbiome differences in indigenous Filipinos with chronic otitis media due to a duplication in the *A2ML1* gene

**DOI:** 10.1186/s40249-016-0189-7

**Published:** 2016-11-01

**Authors:** Regie Lyn P. Santos-Cortez, Diane S. Hutchinson, Nadim J. Ajami, Ma. Rina T. Reyes-Quintos, Ma. Leah C. Tantoco, Patrick John Labra, Sheryl Mae Lagrana, Melquiadesa Pedro, Erasmo Gonzalo d. V. Llanes, Teresa Luisa Gloria-Cruz, Abner L. Chan, Eva Maria Cutiongco-de la Paz, John W. Belmont, Tasnee Chonmaitree, Generoso T. Abes, Joseph F. Petrosino, Suzanne M. Leal, Charlotte M. Chiong

**Affiliations:** 1 Department of Molecular and Human Genetics, Center for Statistical Genetics, Baylor College of Medicine, Houston, TX 77030 USA; 2 Department of Molecular Virology and Microbiology, Alkek Center for Metagenomics and Microbiome Research (CMMR), Baylor College of Medicine, Houston, TX 77030 USA; 3 Philippine National Ear Institute, University of the Philippines Manila – National Institutes of Health (UPM-NIH), Manila, 1000 Philippines; 4 Department of Otorhinolaryngology, University of the Philippines College of Medicine – Philippine General Hospital, Manila, 1000 Philippines; 5 Institute of Human Genetics, UPM-NIH, Manila, 1000 Philippines; 6 Philippine Genome Center, University of the Philippines, Diliman, Quezon City, 1101 Philippines; 7 Departments of Molecular and Human Genetics and Pediatrics, Baylor College of Medicine, Houston, TX 77030 USA; 8 Division of Pediatric Infectious Disease and Immunology, Department of Pediatrics, University of Texas Medical Branch, Galveston, TX 77555 USA; 9 Current affiliation: Department of Otolaryngology, University of Colorado School of Medicine, Aurora, CO 80045 USA; 10 Current address: Illumina, Inc, San Diego, CA 92122 USA

**Keywords:** *A2ML1*, Indigenous population, Microbiome, Middle ear, *Oligella*, Otitis media, Philippines

## Abstract

**Background:**

Previously rare *A2ML1* variants were identified to confer otitis media susceptibility in an indigenous Filipino community and in otitis-prone US children. The goal of this study is to describe differences in the middle ear microbiome between carriers and non-carriers of an *A2ML1* duplication variant that increases risk for chronic otitis media among indigenous Filipinos with poor health care access.

**Methods:**

Ear swabs were obtained from 16 indigenous Filipino individuals with chronic otitis media, of whom 11 carry the *A2ML1* duplication variant. Ear swabs were submitted for 16S rRNA gene sequencing.

**Results:**

Genotype-based differences in microbial richness, structure, and composition were identified, but were not statistically significant. Taxonomic analysis revealed that the relative abundance of the phyla Fusobacteria and Bacteroidetes, and genus *Fusobacterium* were nominally increased in carriers compared to non-carriers, but were non-significant after correction for multiple testing. We also detected rare bacteria including *Oligella* that was reported only once in the middle ear.

**Conclusions:**

These findings suggest that *A2ML1-*related otitis media susceptibility may be mediated by changes in the middle ear microbiome. Knowledge of middle ear microbial profiles according to genetic background can be potentially useful for therapeutic and prophylactic interventions for otitis media and can guide public health interventions towards decreasing otitis media prevalence within the indigenous Filipino community.

**Electronic supplementary material:**

The online version of this article (doi:10.1186/s40249-016-0189-7) contains supplementary material, which is available to authorized users.

## Multilingual abstracts

Please see Additional file 1 for translations of the abstract into the six official working languages of the United Nations.

## Background

Otitis media or middle ear infection is an important public health problem worldwide. In developed countries otitis media remains the top reason for health care visits and antibiotic use among children [1], while incidence and prevalence of otitis media are particularly high in sub-Saharan Africa, Asia and in marginalized communities such as indigenous populations, where the burden of complications due to chronic otitis media (e.g., hearing loss) is mostly felt [2]. Due to high prevalence of chronic suppurative otitis media and its complications in developing countries, it was recently proposed for chronic otitis media to be classified as a neglected tropical disease [3]. While multiple risk factors are associated with otitis media, there is strong evidence that a genetic basis for otitis media susceptibility exists [4, 5].

Recently we identified rare variants in the *A2ML1* gene, which encodes alpha-2-macroglobulin-like 1 protein, as a cause of otitis media susceptibility [6]. A rare *A2ML1* duplication variant c.2478_2485dupGGCTAAAT (p.(Ser829Trpfs*9)) confers susceptibility to otitis media in three European- or Hispanic-American children in Texas, USA and an indigenous Filipino population. This variant co-segregated with different forms of otitis media in a six-generation pedigree within the indigenous Filipino community, which is highly intermarried due to socio-economic segregation and cultural discrimination and has a relatively homogeneous environmental background i.e., poor health care access and hygiene, lack of pneumococcal vaccination, crowded households and swimming in dirty seawater. In this population, known risk factors for otitis media including quantitative age, gender, nutrition and tobacco exposure were not associated with otitis media status [7]. Additionally the three US children who also carry the *A2ML1* duplication variant had early-onset recurrent otitis media that required tympanostomy tube insertion within the first six months of age. The duplication variant occurred within a short haplotype that was common among the indigenous Filipinos and the US children, suggesting a founder variant that is estimated to be 1 800 years old and that may have occurred within the Filipino and US populations through Spanish colonization [6].

Genetic susceptibility to otitis media has been established using family and association studies, however the mechanisms by which genetic variants influence host-bacterial interactions in the middle ear has not been elucidated. Here we show suggestive evidence that carriage of the *A2ML1* duplication variant might influence the middle ear microbial composition of individuals with chronic otitis media, which may explain in part the pathogenic mechanism by which the *A2ML1* duplication variant confers otitis media susceptibility. This study is novel for several reasons: (A) Most microbiome studies for (non-chronic) otitis media used nasopharyngeal and adenoid samples rather than middle ear fluid or swabs, and differences in microbial profiles according to sample source within the head and neck is known [8, 9]. (B) Previously middle ear microbiome studies were performed for chronic otitis media using a single sample and on 11 indigenous Australian children with effusive otitis media [10, 11]. (C) Comparison of microbiome findings according to human host genotype has only been done for a few diseases (e.g., cystic fibrosis, inflammatory bowel disease), but not otitis media [12, 13]. This study also further supports the concept that in the presence of human or host mutation, the complexity of disease patterns can be partly attributed to changes in the microbiome.

## Methods

The study was approved by the: Baylor College of Medicine (BCM) Institutional Review Board and Affiliated Hospitals; the National Commission on Indigenous Peoples, Philippines; and the University of the Philippines Manila Research Ethics Board. Informed consent was obtained from adult participants and parents or guardians of pediatric patients. Participating individuals from an indigenous Filipino community were examined by otoscopy. Previous analyses of multiple risk factors for otitis media established a homogeneous environmental background for this community [7]. Despite high prevalence of otitis media, this community has poor access to health care including vaccinations and antibiotic treatments and has no surgical facilities within the island. In many cases, otitis media either spontaneously resolves with age or results in chronic eardrum perforation with recurrent discharge. Chronically perforated eardrums result in long-term exposure of the middle ear mucosa to the outer ear flora and the environment, e.g., during bathing or swimming in the sea.

From each individual, human genomic DNA from saliva was obtained using Oragene DNA saliva kits (DNA Genotek, Ontario, Canada). DNA samples were Sanger-sequenced for the *A2ML1* duplication variant [6]. From indigenous Filipinos with perforated eardrum(s) due to chronic otitis media, outer ear swabs were collected prior to middle ear swabs, using sterile short polyester-tipped Pur-Wrap swabs (Puritan Medical, Guilford, ME, USA). The outer ear swabs were rubbed gently against outer ear canal skin, then additional swabs were rubbed against middle ear mucosa and/or edges of eardrum perforations and soaked on discharge when present. Each swab specimen was placed directly in the collection tube from the PowerSoil DNA isolation kit (MO BIO Laboratories, Carlsbad, CA, USA), the swab stem cut with sterile scissors, and the collection tube closed, sealed and labeled. All samples were stored in a -20 °C freezer until shipped to BCM on dry ice.

All collected swabs were submitted to the BCM Alkek Center for Metagenomics and Microbiome Research for 16S rRNA gene profiling. Bacterial genomic DNA was isolated from ear swabs using the PowerSoil DNA isolation kit. 16S rRNA gene sequencing methods were adapted from the NIH-Human Microbiome Project. Briefly, the 16S rRNA gene V4 region was amplified by PCR and sequenced on an Illumina MiSeq (2 × 250 bp). The primers (515F and 806R) used for amplification contain adapters for MiSeq sequencing and single-index barcodes allowing PCR products to be pooled and sequenced directly [14, 15]. The read pairs were demultiplexed based on the unique molecular barcodes and filtered at an expected error rate of 0.05. Reads were truncated at first instance of Q5 and then size selected between 252-254 bp. Reads were merged using USEARCH v7.0.1001 [16] using an 8 bp seed sequence, at least 50 bp of overlap, and zero mismatches across the overlap. The 16S rRNA gene sequencing analysis pipeline incorporates phylogenetic and alignment-based approaches to maximize data resolution. 16S rRNA gene sequences were assigned into Operational Taxonomic Units (OTUs) at a similarity cutoff value of 97 % using the UPARSE algorithm [16]. OTUs were mapped at 97 % sequence identity to an optimized version of the SILVA 123 database, containing only the V4 region to determine taxonomy. Abundances were recovered by mapping demultiplexed reads to the UPARSE OTUs. Sequences were normalized to 10 000 sequences per sample.

The microbial sequence data was analyzed for α-diversity, which measures richness, evenness, or diversity of individual samples, and β-diversity, which measures dissimilarity between samples based on phylogenetic distance and taxonomic composition [17]. Because multiple swabs were available per individual, outer ear swabs and middle ear swabs were analyzed separately. For bilateral samples, swabs from the right ear were included in analyses. The PERMANOVA test was applied to β-diversity metrics to ascertain differences in community composition between wildtype individuals and *A2ML1* variant carriers. Differential abundance of taxa was detected by the Mann-Whitney test with false discovery rate (FDR, Benjamini-Hochberg) correction for multiple comparisons (*P <* 0.05 significance threshold). 16S rRNA gene compositional analysis provided a summary of the composition and structure of the bacterial component of the microbiome. Due to our previous findings that aside from the *A2ML1* duplication no other risk factors were associated with otitis media status within the indigenous Filipino community [7], no other variables were used in the analyses.

## Results

Middle and outer ear swabs were obtained from 16 indigenous Filipino individuals (Table 1), 11 of whom were carriers of the *A2ML1* variant. Median age was 9.5 years, and females comprised 62.5 % of study subjects. Half of the individuals with middle ear samples were bilaterally affected. Clinical history and otoscopic findings from follow-up over 2-10 years indicated chronic otitis media for all individuals examined, with the last examination revealing well-defined edges of eardrum perforations ranging from 20 % to near-total perforations. Description of discharge when present varied widely, being copious or scanty with moist middle ear mucosa, purulent or mucoid, foul- or non-foul-smelling. When visualized, the middle ear mucosa was usually thickened. In three ears, granulation tissue was observed. Also two individuals had chronic otitis media in one ear, but acute or effusive otitis media in the other ear. According to local health office records, none of the 16 indigenous Filipinos with chronic otitis media had vaccinations for common pathogens for acute otitis media, i.e., *Streptococcus pneumoniae* or *Haemophilus influenzae*, or recent antibiotic use at time of examination.

**Table 1:** Clinical data and *A2ML1* duplication variant genotypes of indigenous Filipinos with ear swabs

ID	Age (yrs)	Sex	GT	Side	Otoscopic findings
2	10	F	wt	L	20 % perforation and copious yellowish foul-smelling discharge; reddish dull TM
5	10	M	het	B	60-80 % perforation with purulent foul-smelling discharge
7	4	M	het	R	30 % perforation with purulent thick non-foul-smelling discharge
8	24	F	wt	L	20 % perforation, dry
20	6	F	het	R	20 % perforation, scanty non-foul-smelling discharge
33	9	M	wt	B	near-total perforation with thick purulent non-foul-smelling discharge
41	10	F	het	R	70 % perforation with moist middle ear mucosa
45	19	F	het	B ^a^	B: 20 % perforation with yellowish non-foul-smelling discharge: L: granulation tissue
53	14	F	het	B	30-40 % perforation with discharge
62	6	M	hom	R	20 % perforation with non-foul-smelling discharge
63	13	F	hom	B	40-90 % perforation with moist middle ear mucosa
67	6	F	het	L	20 % perforation, dry
71	12	F	wt	L	50 % perforation with moist middle ear mucosa
76	7	M	wt	B ^b^	L: 30 % perforation with discharge; R: intact but retracted TM
89	4	F	het	B ^b^	R: foul-smelling discharge; L: dull, bulging erythematous TM
92	7	M	het	B	filled with granulation tissue and foul-smelling discharge

*GT* genotype, *F* female, *M* male, *wt* wildtype, *het* heterozygous, *hom* homozygous, *L* left, *R* right, *B* bilateral, *TM* tympanic membrane

^a^ID 45 has chronic otitis media bilaterally but middle ear sample is available only for the right ear

^b^Perforation only in one ear, for which a middle ear sample was obtained

Middle and outer ear swabs were available from a total of 21 ears with chronic otitis media from 16 indigenous Filipinos (Table 1; Additional file 2: Figure S1). The median DNA concentration per ear swab was 3.0 ηg/μl (maximum 92.5 ηg/μl), indicating that these samples contain high bacterial biomass. For five individuals with bilateral samples, middle ear microbial profiles were not different between ears (Additional file 3: Figure S2), thus for these five individuals only the right ear was included in comparisons by genotype. When comparing middle ears of wildtype versus *A2ML1-*positive individuals, observed OTUs were not different (Additional file 2: Figure S1A). The mean Shannon diversity index was higher in middle ears of carriers, however the difference was not statistically significant (Additional file 2: Figure S1B). Similar plots were also non-significant for the outer ears (Additional file 4: Figure S3). The β-diversity analyses for middle ear swabs were not statistically significant between carriers and non-carriers (Additional file 2: Figures S1C and S1D). Lack of significant results for these metrics may be largely due to the small sample size.

**Fig. 1:**
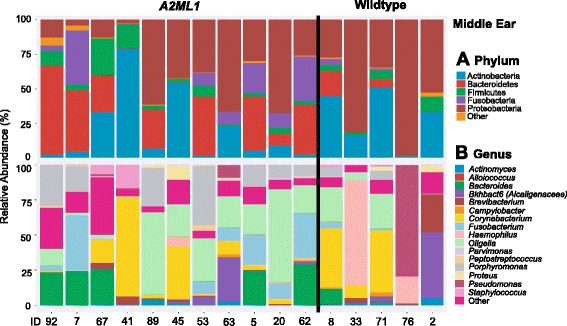
Relative abundance of middle ear bacterial taxa according to *A2ML1* genotype. **a** By phylum; **b** by genus

At phylum level, relative taxa abundance profiles for the middle ear differed by genotype, with more Fusobacteria and Bacteroidetes in carriers of the *A2ML1* variant than in non-carriers (Table 2, Fig. 1a). At genus level, *Fusobacterium* was also greater in carriers (Table 3, Fig. 1b). The higher relative abundance for these taxa was nominally significant in carriers (*P* ≤ 0.05), but non-significant after correction for multiple testing which is not unexpected given the small sample size. Based on identified genera the composition of the middle ear microbiome in the *A2ML1* variant carriers differed from non-carriers due to higher relative abundance in *Fusobacterium, Porphyromonas, Peptostreptococcus, Parvimonas* and *Bacteroides* (Table 3, Fig. 1b), while median frequencies for *Alloiococcus, Staphylococcus, Proteus* and *Haemophilus* were greater in wildtype middle ears (Table 3). These genera show concordance with identified phyla, e.g., higher relative abundance of the phyla Fusobacteria and Bacteroidetes in middle ears of *A2ML1-*positive individuals due to greater median frequencies for genera *Fusobacterium* and *Porphyromonas/Bacteroides,* respectively (Fig. 1; Table 3). On the other hand, non-significant increases in relative abundance were detected for the phyla *Proteobacteria* (i.e., due to *Proteus, Haemophilus, Alcaligenaceae* or *Pseudomonas*) and *Actinobacteria* (i.e., *Brevibacterium, Actinomyces* or *Corynebacterium*) in middle ears of non-carriers (Tables 2 and 3).

**Table 2:** Bacterial phyla in middle ears of indigenous Filipinos with chronic otitis media

Phylum	Overall median %	*A2ML1+* median %	Wildtype median %	Mann- Whitney *p*	FDR-adjusted *p* ^a^
Fusobacteria	2.72	9.15	0.04	0.04	0.24
Bacteroidetes	13.51	28.03	0.16	0.05	0.24
Proteobacteria	36.31	29.59	52.75	0.15	0.44
Actinobacteria	12.40	6.98	33.28	0.51	0.74
Firmicutes	3.96	3.94	4.30	0.83	0.83

^a^False discovery rate (FDR)

**Table 3:** Middle ear bacterial genera with ≥0.1 % difference in relative abundance by genotype

Phylum	Genus	Overall median %	*A2ML1+* median %	Wildtype median %	Mann- Whitney *p* ^a^
Fusobacteria	*Fusobacterium*	2.72	9.15	0.04	0.05
Firmicutes	*Alloiococcus*	0	0	0.24	0.06
Bacteroidetes	*Porphyromonas*	6.22	9.00	0.06	0.07
Firmicutes	*Staphylococcus*	0.03	0.01	0.11	0.11
Firmicutes	*Peptostreptococcus*	0.05	0.19	0.01	0.14
Firmicutes	*Parvimonas*	0.19	0.21	0.01	0.21
Proteobacteria	*Proteus*	0.10	0	0.16	0.24
Bacteroidetes	*Bacteroides*	0.36	0.69	0.08	0.30
Proteobacteria	*Haemophilus*	0.17	0.07	0.41	0.31
Proteobacteria	*Oligella*	20.48	21.07	5.33	0.38
Actinobacteria	*Brevibacterium*	0.41	0.22	0.66	0.40
Actinobacteria	*Actinomyces*	0.73	0.50	1.11	0.44
Proteobacteria	*Bkhbact6 (Alcaligenaceae)*	0.51	0.44	0.65	0.61
Proteobacteria	*Campylobacter*	0.19	0.29	0	0.73
Proteobacteria	*Pseudomonas*	0.06	0.03	0.15	0.77
Actinobacteria	*Corynebacterium*	3.78	3.74	8.62	1

^a^All FDR-adjusted *p*-values equal 1

**Table 4:** Bacterial phyla in outer ears of indigenous Filipinos with chronic otitis media

Phylum	Overall median %	*A2ML1+* median %	Wildtype median %	Mann- Whitney *p*	FDR-adjusted *p* ^a^
Bacteroidetes	2.97	32.25	0.05	0.009	0.07
Fusobacteria	1.56	3.11	0	0.04	0.16
Proteobacteria	34.89	34.56	75.20	0.27	0.36
Firmicutes	3.49	3.63	0.59	0.44	0.50
Actinobacteria	15.07	11.97	24.39	0.91	0.91

**Table 5:** Outer ear bacterial genera with >0.1 % difference in relative abundance by genotype

Genus	Overall median %	*A2ML1+* median %	Wildtype median %	Mann- Whitney *p* ^a^	FDR-adjusted *p*
*Porphyromonas*	1.69	7.62	0.02	0.01	0.30
*Fusobacterium*	1.56	3.11	0	0.04	0.39
*Haemophilus*	0.42	0.23	9.61	0.04	0.39
*Parvimonas*	0.11	0.18	0	0.06	0.45
*Bacteroides*	0.25	1.26	0.03	0.09	0.53
*Campylobacter*	0.08	0.18	0	0.11	0.53
*Peptostreptococcus*	0.06	0.19	0.01	0.15	0.54
*Proteus*	0.19	0.03	0.50	0.28	0.63
*Peptoniphilus* ^a^	0.17	0.17	0.07	0.32	0.65
*Corynebacterium*	7.83	3.81	12.00	0.38	0.71
*Pseudoclavibacter* ^b^	1.56	0.66	2.13	0.38	0.71
*Oligella*	22.88	27.23	6.85	0.44	0.75
*Bkhbact6 (Alcaligenaceae)*	0.62	0.83	0.21	0.58	0.75
*Alloiococcus*	0.04	0	0.11	0.59	0.75
*Dietzia* ^b^	0.15	0.01	0.24	0.60	0.75
*Brevibacterium*	0.95	1.17	0.72	0.66	0.78

Phylum information: ^a^ *Firmicutes;* ^b^ *Actinobacteria*

For outer ears the unweighted UniFrac principal coordinate analysis was significant by genotype although weighted UniFrac analysis was non-significant (Additional file 4: Figure S3). The bacterial taxa identified in the outer ears were similar to middle ear bacteria (Tables 4, 5, Additional file 2: Figure S1 and Additional file 4: Figure S3). Overall these findings suggest similarity in middle and outer ear bacteria due to cross-contamination across chronically perforated eardrums. This is further supported by the lack of detection of Bacteroidetes and/or Fusobacteria in previous microbiome studies of the external auditory canals of healthy individuals [18, 19].

## Discussion

These microbiome results provide some insight into the effect of the *A2ML1* duplication variant on otitis media susceptibility [6]. Even though α- and β-diversity measurements were not significantly different in *A2ML1* variant carriers versus non-carriers, there is suggestive evidence that carriage of the *A2ML1* variant and the expected loss-of-function of A2ML1 protein might favor survival and growth of specific bacterial taxa. At genus level, *Alloiococcus* was among the top three bacteria identified from the middle ear, although at very low frequency (Table 3). *Alloiococcus otitidis* was initially discovered in middle ear fluid [20]. On the other hand, *Fusobacterium* and *Porphyromonas* had greater relative abundance in *A2ML1* variant carriers compared to non-carriers at ~9 % median frequency in the middle ear (Table 3). The species *Fusobacterium nucleatum* and *Porphyromonas gingivalis* are less commonly isolated in chronic otitis media [21] but are known to proliferate in oropharyngeal infections [21] or progressive gingivitis and periodontitis which are mucosal diseases in the oral cavity [22]. During infection, these two bacteria along with host inflammatory cells release proteases that degrade mucosal matrix and basement membrane, and of these two bacteria, *P. gingivalis* can produce higher proteolytic activity and elicit a more virulent host response [23]. The protein structure of A2ML1 is highly homologous to the protease inhibitor alpha-2-macroglobulin (A2M), and we previously hypothesized that A2ML1 and A2M might have overlapping protective roles within the middle ear by preventing undue mucosal damage from either bacterial or inflammatory proteases [6]. Interestingly, there is evidence that human A2M efficiently inhibits some cysteine proteases produced by *P. gingivalis,* but another homologous macroglobulin is required to trap all proteinase forms and control proteolytic activity due to *P. gingivalis* [24]. A defect in macroglobulin function in the middle ear mucosa may favor infection with *Porphyromonas* or *Fusobacterium* through unregulated bacterial protease activity.

While *Porphyromonas* and *Fusobacterium* were previously isolated in cultures of ear discharge from chronic otitis media [21, 25], these bacterial groups that are more abundant in the *A2ML1-*variant carriers are quite different from major bacterial isolates from chronic middle ear discharge from the general Filipino population, e.g., *Staphylococcus, Pseudomonas, Proteus* [26]. Our microbiome results are also different from a previous microbiome study which largely detected *Pseudomonas* from middle ear fluid of a US child with chronic otitis media [10]. In another microbiome study of middle ear fluid from indigenous Australian children with effusive otitis media, the top species detected were *Alloiococcus otitidis, Haemophilus influenzae* and *Streptococcus sp.* [11] which are more frequently detected in non-chronic forms of otitis media i.e., acute or effusive otitis media [27]. Note however that *Haemophilus influenzae* has also been identified in chronic otitis media and is known to form biofilm in the middle ear [28]. Although the uniqueness of the bacterial profiles in indigenous Filipinos vs. other populations may be attributed to differences in sampling and detection techniques, type of otitis media and community setting, the differences in diversity and abundant taxa by genotype as described here were detected while comparing indigenous Filipino individuals only. All the indigenous Filipinos studied were diagnosed with chronic otitis media, had clinical history of several years of inadequately treated chronic ear discharge, and were from the same homogeneous community, providing supportive evidence that the microbial profile changes are more likely due to genotype. Moreover the finding of known oral cavity pathogens as the predominant middle ear bacteria in *A2ML1* variant carriers begs further study of a potential link between oral health and otitis media status in these individuals.

For the indigenous Filipino community, microbiome technologies have high utility given the lack of access to laboratory facilities including microbial culture. The identification of bacteria that were rarely or never reported in middle ears also testifies to the utility of 16S rRNA gene profiling in detecting bacteria that may be missed in culture studies. *Oligella* was reported only once in ear discharge [29] while the OTU BkhBact6 (Alcaligenaceae), which was classified to genus GKS98 freshwater group of the class Betaproteobacteria, has only been seen in environmental samples [30]. Bkhbact6 (Alcaligenaceae) had higher relative abundance in the middle ears of wildtype individuals (Table 3). *Oligella* was highly abundant in both middle and outer ears and was occasionally the predominant genus in the middle ear swabs (Table 3, Fig. 1b). In addition, the relative abundance of *Oligella* was greater than that of *Corynebacterium,* which has been isolated from middle and outer ears but considered debatable in terms of pathogenic potential [31]. Although *Oligella* sp. is known as a commensal in the genitourinary tract, it was previously isolated from deep tissue infection and might also be considered an opportunistic pathogen [29]. Currently the paucity of literature on *Oligella* and Bkhbact6 (Alcaligenaceae) precludes conclusion if these bacteria are middle ear pathogens, commensals or contaminants.

## Conclusions

Overall our findings suggest that the *A2ML1* duplication variant may induce changes in the middle ear microbiome. It should be stressed that the study was designed to be exploratory rather than associative, therefore these findings are descriptive in nature and require confirmation using a larger number of subjects. Nevertheless, this study highlights the importance of host genotype-bacterial interactions in understanding the pathophysiology of common infectious diseases such as otitis media. Specifically, within the indigenous Filipino community, knowledge of middle ear microbial profiles according to genetic background can be potentially useful for future therapeutic and prophylactic interventions, such as antibiotic use and vaccination that includes coverage against *Haemophilus* which was detected by 16S rRNA sequencing.

## Additional files

Additional file 1: Multilingual abstracts in the six official working languages of the United Nations. (PDF 763 kb)
Additional file 2: Figure S1. Middle ear microbial profiles of indigenous Filipinos with chronic otitis media. All panels compare carriers with non-carriers of the *A2ML1* duplication variant. Panel description: (A) α-diversity by observed OTUs; (B) α-diversity by the Shannon diversity index; (C) β-diversity from unweighted UniFrac principal coordinate analysis; (D) β-diversity from weighted UniFrac principal coordinate analysis. (PDF 1019 kb)
Additional file 3: Figure S2. Comparison of right and left ears from five individuals with bilateral ear samples. Plots comparing the most abundant phyla and genera in the outer and middle ears show very similar profiles between right and left ears. *Blue bars* represent taxa that are more abundant in the right ear, *green bars* for the left ear. Only phylum Firmicutes (*P* = 0.02) and genus *Alcaligenaceae* which belongs to phylum Proteobacteria (*P* = 0.02) were significantly different between right and left outer ear swabs from these five individuals, however *p-*values were non-significant for all comparisons after correction for multiple testing. For the five individuals with bilateral samples, only right ear swabs were included in microbial analyses by genotype. (PDF 750 kb)
Additional file 4: Figure S3. Outer ear microbial profiles by genotype. Panel description as in Additional file 2: Figure S1. (PDF 783 kb)

## Abbreviations

OTU: Operational taxonomic units
PCR: Polymerase chain reaction
