# Supplementary material for: Middle ear microbiome differences in indigenous Filipinos with chronic otitis media due to a duplication in the A2ML1 gene
Source: Infect Dis Poverty. 2016 Nov 1;5:97. doi: 10.1186/s40249-016-0189-7 (PMC5088646; doi:10.1186/s40249-016-0189-7)
Supplement: Additional file 1: — Multilingual abstracts in the six official working languages of the United Nations. (PDF 763 kb) [file 40249_2016_189_MOESM1_ESM.pdf]

Translation of the abstract into the six official working languages of the United Nations

اختلافات الميكروبيوم بالأذن الوسطى للسكان من أصل فيليبيني المصابون بالتهاب الأذن الوسطى المزمن بسبب تضاعف جينة *A2ML1*

ريجي لين ب. سانتوس-كورتوز، ديان س. هيوتشينسون، ناديم ج. أجامي، ما. رينا ت.رايس-كوينتوس، ما. لياه س. تانتاكو، باتريك جون لابرا، شاريل ماي لاغرانا، مالكيادازا بادرو، أراسمو غونزالو د.ف. للاناس، تيريزا لويزا غلوريا-كروز، أبنر ل.شان، إيفا ماريا كويتينغكو-دي لا باز، جون و. بالمونت، تازنيي كونماتريي، غناروزو ت.عباس، جوزيف ف. باتروزينو، سوزان م. ليل، شارلوت م. شيونغ

ملخص

تقديم عام: تم تحديد متغيرات *A2ML1* النادرة سابقا، للبحث عن مدى سهولة إصابة السكان ذو الأصل الفيليبيني وأطفال الولايات المتحدة بالتهاب الأذن الوسطى. تهدف هذه الدراسة إلى وصف اختلافات الميكروبيوم بالأذن الوسطى بين حاملي جينة *A2ML1* وغير الحاملين لها التي تزيد من مخاطر التهاب الأذن الوسطى المزمن بين السكان ذو الأصل الفيليبيني الذين يفتقرون لرعاية صحية جيدة.

الأساليب المستخدمة: تم الحصول على مسحات الأذن من 16 شخصًا ذو أصل فليبيني ممن يُعانون من التهاب الأذن الوسطى المزمن، من بينهم 11 مصابًا حاملا لجينة *A2ML1*. تم خُضوع مسحات الأذن لعملية التسلسل الجيني 16S rRNA.

النتائج: تم تحديد الفوارق القائمة على الخصائص الوراثية على مستوى تعدد الميكروبات وهيكلتها وتركيبتها، لكن لم تكن ذات دلالة إحصائية. كشف التحليل التصنيفي أن الوفرة النسبية المرتبطة بشعبة *Fusobacteria* و *Bacteroidetes* وجنس *Fusobacterium* قد شهدت ارتفاعا بالنسبة للحاملين مقارنة بأولئك غير الحاملين للجينة، إلا أنها لم تكن مهمة بعد التصحيح نتيجة الخضوع لعديد الاختبارات. كما اكتشفنا أيضا بكتريا نادرة بما في ذلك *Oligella* التي تم الإبلاغ عن وجودها بالأذن الوسطى مرة واحدة.

الخاتمة: تُشير هذه النتائج إلى أن سهولة الإصابة بالتهاب الأذن الوسطى المرتبط بجينة *A2ML1* يمكن أن ينتج عن تغيرات مكروبيوم الأذن الوسطى. يمكن الاستفادة من معرفة خاصيات ميكروبيوم الأذن الوسطى استنادا على الخلفية الوراثية على مستوى المداخلات العلاجية والوقائية فيما يخص التهاب الأذن الوسطى وعلى مستوى توجيه المداخلات المتعلقة بالصحة العمومية بهدف الحد من انتشار هذا المرض بين السكان ذو الأصل الفيليبيني.

Translated from English version into Arabic by Zeineb TRABELSI, through

TRANSLATORS
WITHOUT BORDERS

基于 *A2ML1* 基因复制研究菲律宾土著慢性中耳炎患者中耳道菌群的差异

Regie Lyn P. Santos-Cortez, Diane S. Hutchinson, Nadim J. Ajami, Ma. Rina T. Reyes-Quintos, Ma. Leah C. Tantoco, Patrick John Labra, Sheryl Mae Lagrana, Melquiadesa Pedro, Erasmo Gonzalo d.V. Llanes, Teresa Luisa Gloria-Cruz, Abner L. Chan, Eva Maria Cutiongco-de la Paz, John W. Belmont, Tasnee Chonmaitree, Generoso T. Abes, Joseph F. Petrosino, Suzanne M. Leal, Charlotte M. Chiong

摘要

引言：先前研究在菲律宾土著人和中耳炎易感美国儿童人群中发现罕见的 *A2ML1* 基因变异体与中耳炎易感性有关。本研究旨在阐述在缺乏卫生保健的菲律宾土著人群中 *A2ML1* 基因复制变异体携带者与非携带者的中耳道菌群的差异导致的慢性中耳炎的风险增加。

方法：获取 16 例菲律宾土著中耳炎患者的耳拭子，其中 11 例携带 *A2ML1* 基因复制变异体。16 份耳拭子样本均进行 16S rRNA 基因测序。

结果：根据基因型差异确定了患者中耳道菌群丰度、结构和组成均不同，但差异无统计学意义。分类分析结果显示，耳拭子中含有大量的梭杆菌门(Fusobacteria)和拟杆菌门(Bacteroidetes)细菌，其中 A2ML1 基因携带者比非携带者的梭形杆菌属(*Fusobacterium*)种类更多，但经多重检验校正后二者间的差异无统计学意义。本研究还检测到罕见细菌，如仅在中耳中报道过一次的寡源杆菌（*Oligella*）。

结论：本研究结果显示 A2ML1 基因相关的中耳炎易感性可能是由中耳道菌群差异导致的。根据遗传信息了解中耳道菌群情况可用于中耳炎的治疗和预防干预，也可为减少菲律宾土著人群的中耳炎发病提供预防性干预措施的制定依据。

Translated from English version into Chinese by Hong Tu, edited by Pin Yang

CHINESE CENTER FOR DISEASE CONTROL AND PREVENTION
NATIONAL INSTITUTE OF PARASITIC DISEASES

Différences dans le microbiome de l'oreille moyenne chez des indigènes des Philippines souffrant d'otite moyenne chronique due à une duplication dans le gène *A2ML1*

Regie Lyn P. Santos-Cortez, Diane S. Hutchinson, Nadim J. Ajami, Ma. Rina T. Reyes-Quintos, Ma. Leah C. Tantoco, Patrick John Labra, Sheryl Mae Lagrana, Melquiadesa Pedro, Erasmo Gonzalo d.V. Llanes, Teresa Luisa Gloria-Cruz, Abner L. Chan, Eva Maria Cutiongco-de la Paz, John W. Belmont, Tasnee Chonmaitree, Generoso T. Abes, Joseph F. Petrosino, Suzanne M. Leal, Charlotte M. Chiong

Résumé
Contexte: Des variantes d'*A2ML1* rares jusqu'ici ont été identifiées comme des facteurs de susceptibilité à l'otite moyenne dans un groupe autochtone des Philippines et chez des enfants prédisposés aux otites aux États-Unis. Le but de notre étude est de décrire les différences de microbiome de l'oreille moyenne entre les sujets porteurs et non porteurs d'une variante de duplication d'*A2ML1* augmentant le risque d'otite moyenne chronique chez des indigènes des Philippines qui ne disposent pas d'un bon accès aux soins.
Méthodes: Des frottis du conduit auriculaire ont été recueillis chez 16 indigènes philippins souffrant d'otite moyenne chronique dont 11 présentaient la variante de duplication d'*A2ML1*. Ces frottis ont fait l'objet d'un séquençage des gènes ARNr 16s.
Résultats: À partir du génotype, nous avons identifié des différences de richesse, de structure et de composition du microbiome, qui n'étaient toutefois pas significatives. L'analyse taxonomique a révélé une abondance relative des phylums Fusobacteria et Bacteroidetes ainsi que du genre *Fusobacterium* nominalement plus élevée chez les porteurs du variant que chez les non-porteurs, mais cette différence n'était pas significative après correction pour tenir compte des tests multiples. Nous avons également trouvé des bactéries rares, comme *Oligella* qui n'avait été rapportée qu'une fois dans l'oreille moyenne.
Conclusions: Nos résultats suggèrent que la susceptibilité à l'otite moyenne liée à *A2ML1* est peut-être médiée par des changements du microbiome de l'oreille moyenne. La connaissance des profils microbiens de l'oreille moyenne en fonction du contexte génétique est potentiellement utile pour la prophylaxie et le traitement de l'otite moyenne et peut orienter des interventions de santé publique visant à réduire la prévalence de cette pathologie parmi les peuples indigènes des Philippines.

Translated from English version into French by Suzanne Assenat, through

TRANSLATORS
WITHOUT BORDERS

Различия микробиома среднего уха у исконных филиппинцев с хроническим отитом среднего уха из-за дупликации в гене *A2ML1*

Реджи Лин П. Сантос-Кортез, Диан С. Хатчинсон, Надим Дж. Аями, Ма. Рина Т. Рейс-Квинтос, Ма. Лия С. Тантоко, Патрик Джон Лабра, Шерил Мэй Лаграна, Мельквиадеса Педро, Эразмо Гонзало де В. Йанес, Тереза Луиса Глория-Круз, Абнер Л. Чан, Ева Мария Кутнгко-де-ла-Паз, Джон У. Бельмонт, Тасни Чономайтри, Генеросо Т. Абес, Джозеф Ф. Петросино, Сюзан М. Леаль, Шарлотт М. Чионг

Краткое изложение
История вопроса: Были определены вариации гена *A2ML1*, которые до этого были редки, для подтверждения предрасположенности к отиту среднего уха среди исконных филиппинцев и детей в США с частыми отитами. Целью данного исследования является описание различий в микробиоме среднего уха между носителями и неносителями вариации гена *A2ML1* с дупликацией, повышающей риск хронического отита среднего уха у исконных филиппинцев с ограниченным доступом к медицинскому обслуживанию.
Методы: Были собраны ушные мазки 16 коренных филиппинцев с хроническим отитом среднего уха, из которых у 11 наблюдалась дупликация в гене *A2ML1*. Ушные мазки были сданы на генетическое секвенирование 16S rRNA.
Результаты: Были определены основанные на генотипе различия в микробной насыщенности, структуре и составе, но они не были статистически значимыми. Таксономический анализ выявил относительное обилие типов Fusobacteria и Bacteroidetes, а вид *Fusobacterium* был незначительно выше у носителей в сравнении с неносителями, но после поправки на множественное тестирование это не было значимым. Мы также обнаружили редкие бактерии, включая *Oligella*, которые были найдены в среднем ухе всего один раз.

Заключение: Данные результаты предполагают, что на предрасположенность к отиту среднего уха, связанную с *A2ML1*, можно воздействовать изменениями в микробиоме среднего уха. Информация о микробных профилях среднего уха, связанных с генетическим фоном, может быть полезной для терапевтических и профилактических вмешательств при отите, а также направлять здравоохранительные действия, направленные на снижение частоты отита среднего уха среди исконного филиппинского населения.

Translated from English version into Russian by Elena McDonnell, through

Diferencias en el microbioma del oído medio en los filipinos indígenas con otitis media crónica debido a una duplicación en el gen *A2ML1*

Regie Lyn P. Santos-Cortez, Diane S. Hutchinson, Nadim J. Ajami, Ma. Rina T. Reyes-Quintos, Ma. Leah C. Tantoco, Patrick John Labra, Sheryl Mae Lagrana, Melquiadesa Pedro, Erasmo Gonzalo d.V. Llanes, Teresa Luisa Gloria-Cruz, Abner L. Chan, Eva Maria Cutiongco-de la Paz, John W. Belmont, Tasnee Chonmaitree, Generoso T. Abes, Joseph F. Petrosino, Suzanne M. Leal, Charlotte M. Chiong

Resumen

Antecedentes: Anteriormente se identificaron que variantes raras de A2ML1 conferían susceptibilidad a la otitis media en una comunidad filipina indígena y en los niños estadounidenses propensos a la otitis. El objetivo de este estudio es describir las diferencias en el microbioma del oído medio entre portadores y no portadores de una variante de la duplicación de A2ML1 que aumenta el riesgo de otitis media crónica entre los indígenas filipinos con escaso acceso a la salud.
Métodos: Se obtuvieron hisopos para los oídos de 16 individuos filipinos indígenas con otitis media crónica, de los cuales 11 llevan la variante de duplicación de A2ML1. Los hisopos para los oídos se sometieron a secuenciación del gen 16S rRNA.
Resultados: Se identificaron diferencias en función del genotipo en la riqueza microbiana, estructura y composición, pero no fueron estadísticamente significativas. El análisis taxonómico reveló que la abundancia relativa de los filos Fusobacteria y Bacteroidetes y el género Fusobacterium se incrementaron nominalmente en los portadores en comparación con los no portadores, pero no fueron significativos después de la corrección para múltiples pruebas. También se detectaron bacterias raras incluyendo Oligella que se informó solo una vez en el oído medio.
Conclusiones: Estos hallazgos sugieren que la susceptibilidad a la otitis media relacionada con A2ML1 puede ser mediada por los cambios en el microbioma del oído medio. El conocimiento de los perfiles microbianos del oído medio, según los antecedentes genéticos, puede ser potencialmente útil para las intervenciones terapéuticas y profilácticas para otitis media y puede orientar las intervenciones de salud pública hacia la disminución de la prevalencia de otitis media dentro de la comunidad indígena filipina.

Translated from English version into Spanish by Susana Rosselli, through
