# Supplementary figures and images for: Middle ear microbiome differences in indigenous Filipinos with chronic otitis media due to a duplication in the A2ML1 gene
Source: Infect Dis Poverty. 2016 Nov 1;5:97. doi: 10.1186/s40249-016-0189-7 (PMC5088646; doi:10.1186/s40249-016-0189-7)

## Middle Ear

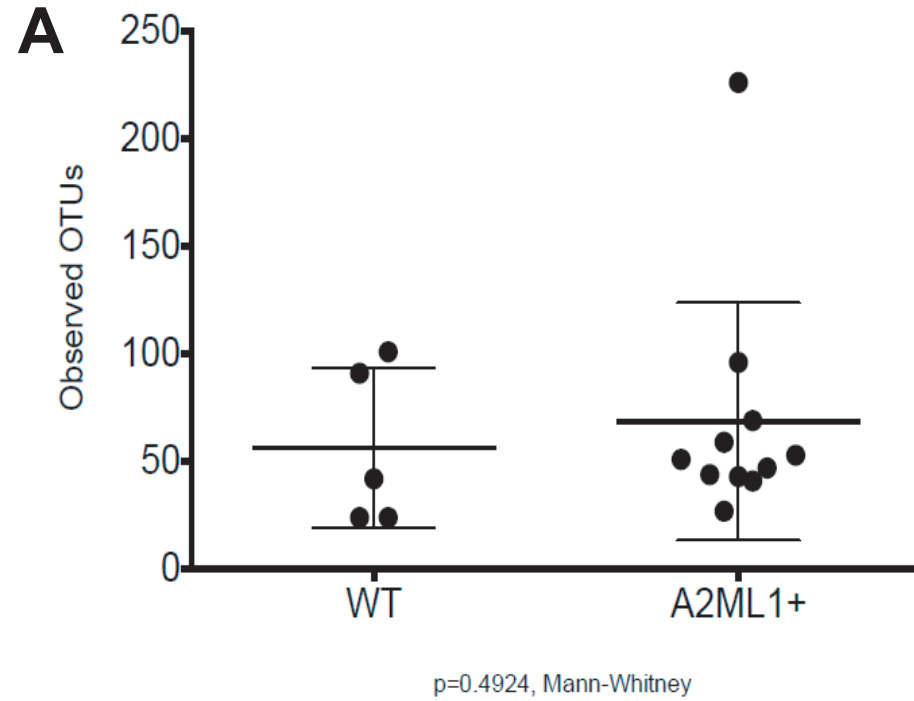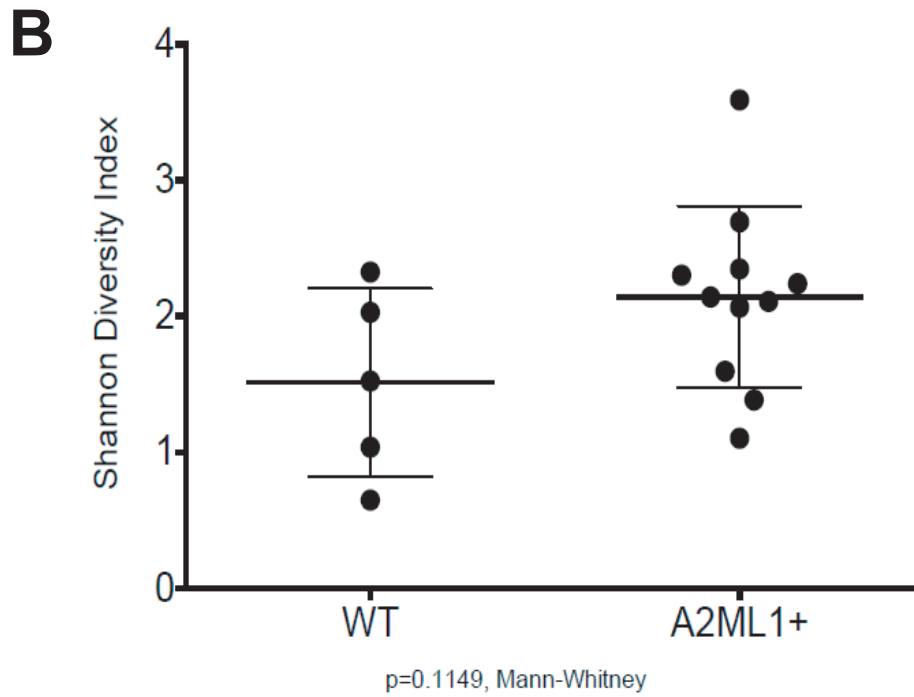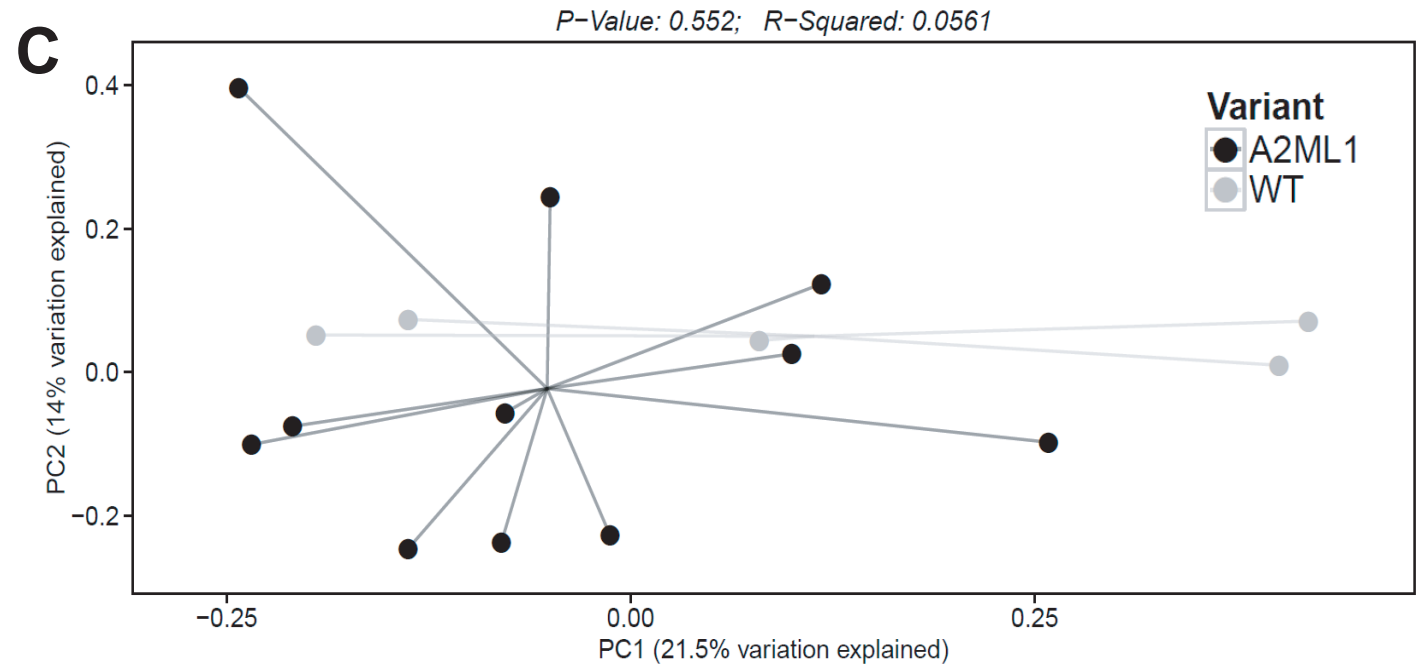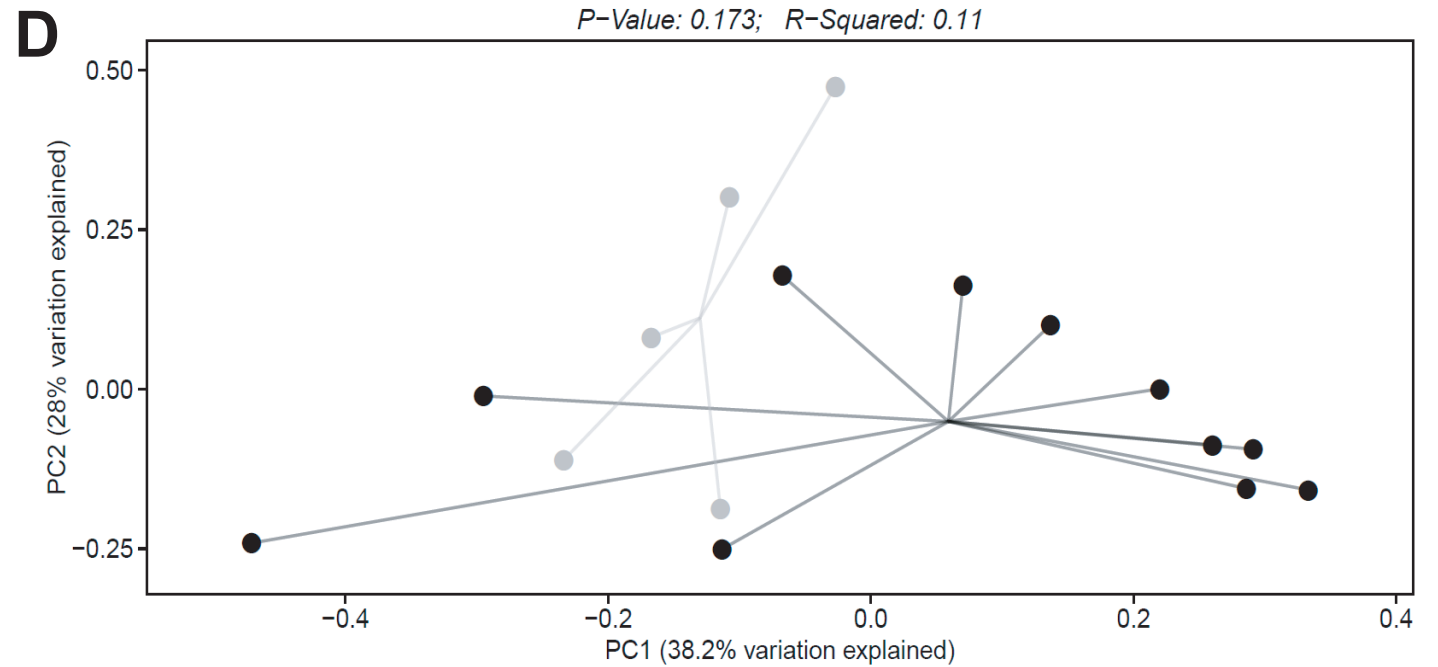

Supplement: Additional file 2: Figure S1. — Middle ear microbial profiles of indigenous Filipinos with chronic otitis media. All panels compare carriers with non-carriers of the A2ML1 duplication variant. Panel description: (A) α-diversity by observed OTUs; (B) α-diversity by the Shannon diversity index; (C) β-diversity from unweighted UniFrac principal coordinate analysis; (D) β-diversity from weighted UniFrac principal coordinate analysis. (PDF 1019 kb) [file 40249_2016_189_MOESM2_ESM.pdf]

## Outer Ear

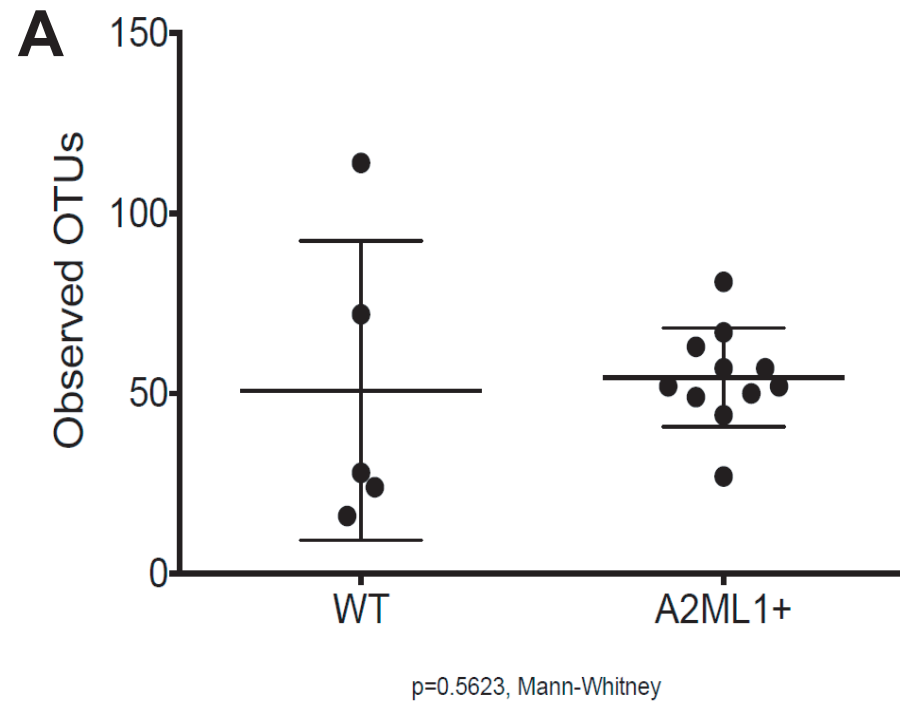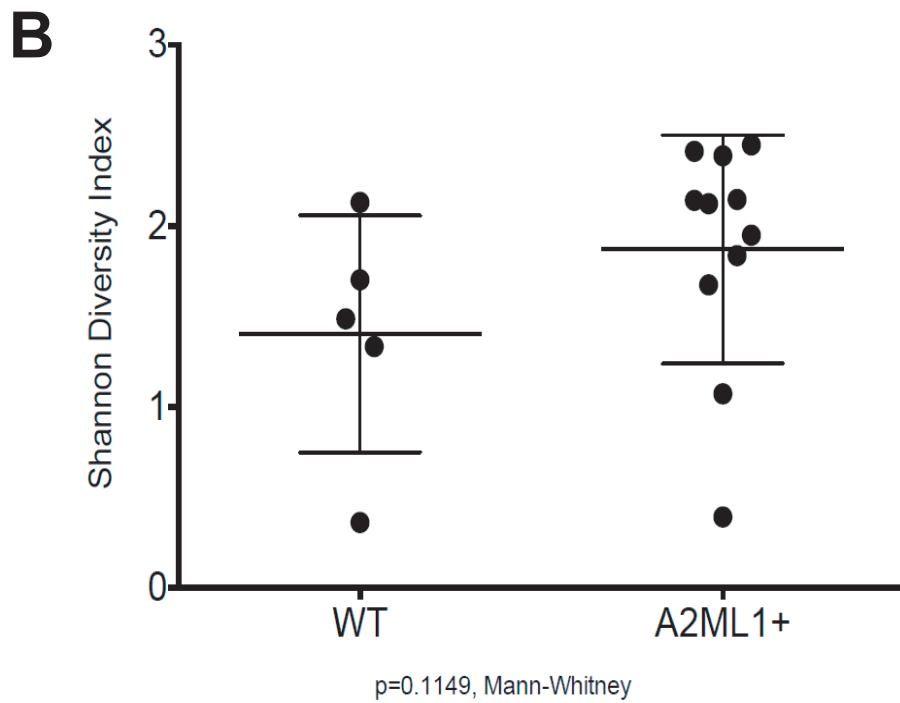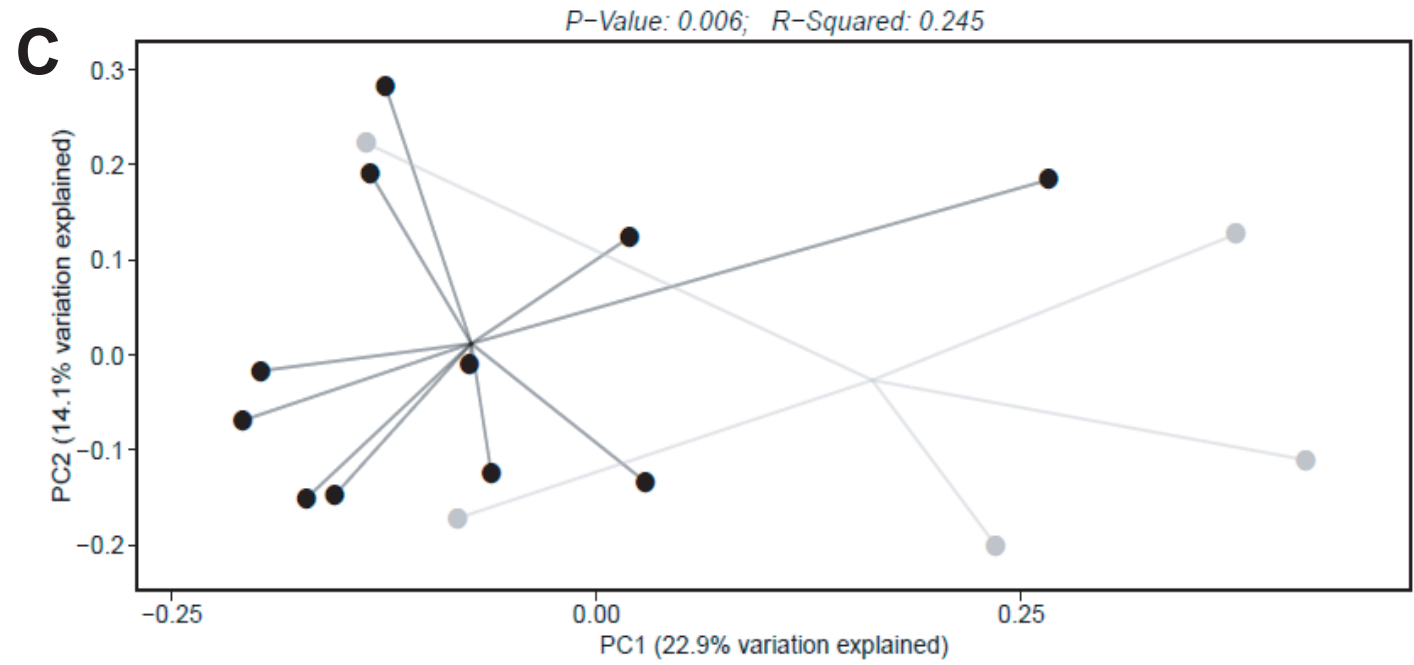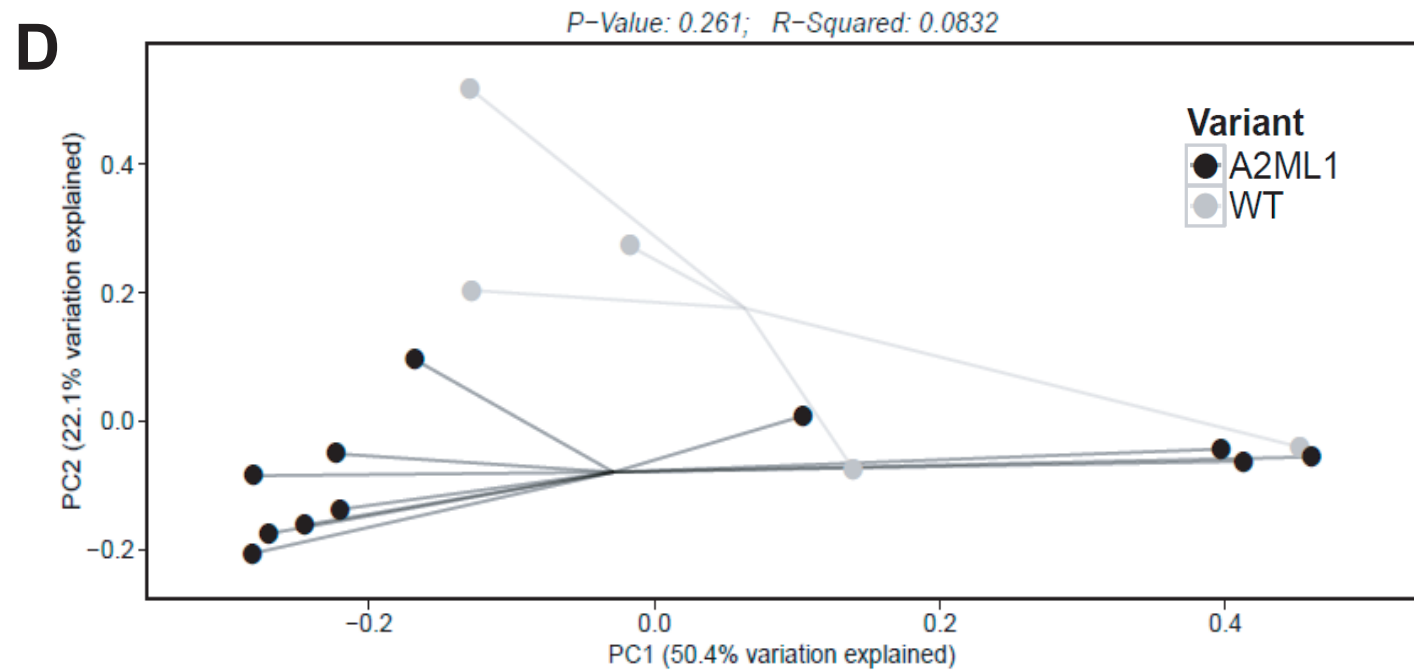

Supplement: Additional file 4: Figure S3. — Outer ear microbial profiles by genotype. Panel description as in Additional file 2: Figure S1. (PDF 783 kb) [file 40249_2016_189_MOESM4_ESM.pdf]
