# Supplementary material for: Middle ear microbiome differences in indigenous Filipinos with chronic otitis media due to a duplication in the A2ML1 gene
Source: Infect Dis Poverty. 2016 Nov 1;5:97. doi: 10.1186/s40249-016-0189-7 (PMC5088646; doi:10.1186/s40249-016-0189-7)
Supplement: Additional file 3: Figure S2. — Comparison of right and left ears from five individuals with bilateral ear samples. Plots comparing the most abundant phyla and genera in the outer and middle ears show very similar profiles between right and left ears. Blue bars represent taxa that are more abundant in the right ear, green bars for the left ear. Only phylum Firmicutes (P = 0.02) and genus Alcaligenaceae which belongs to phylum Proteobacteria (P = 0.02) were significantly different between right and left outer ear swabs from these five individuals, however p-values were non-significant for all comparisons after correction for multiple testing. For the five individuals with bilateral samples, only right ear swabs were included in microbial analyses by genotype. (PDF 750 kb) [file 40249_2016_189_MOESM3_ESM.pdf]

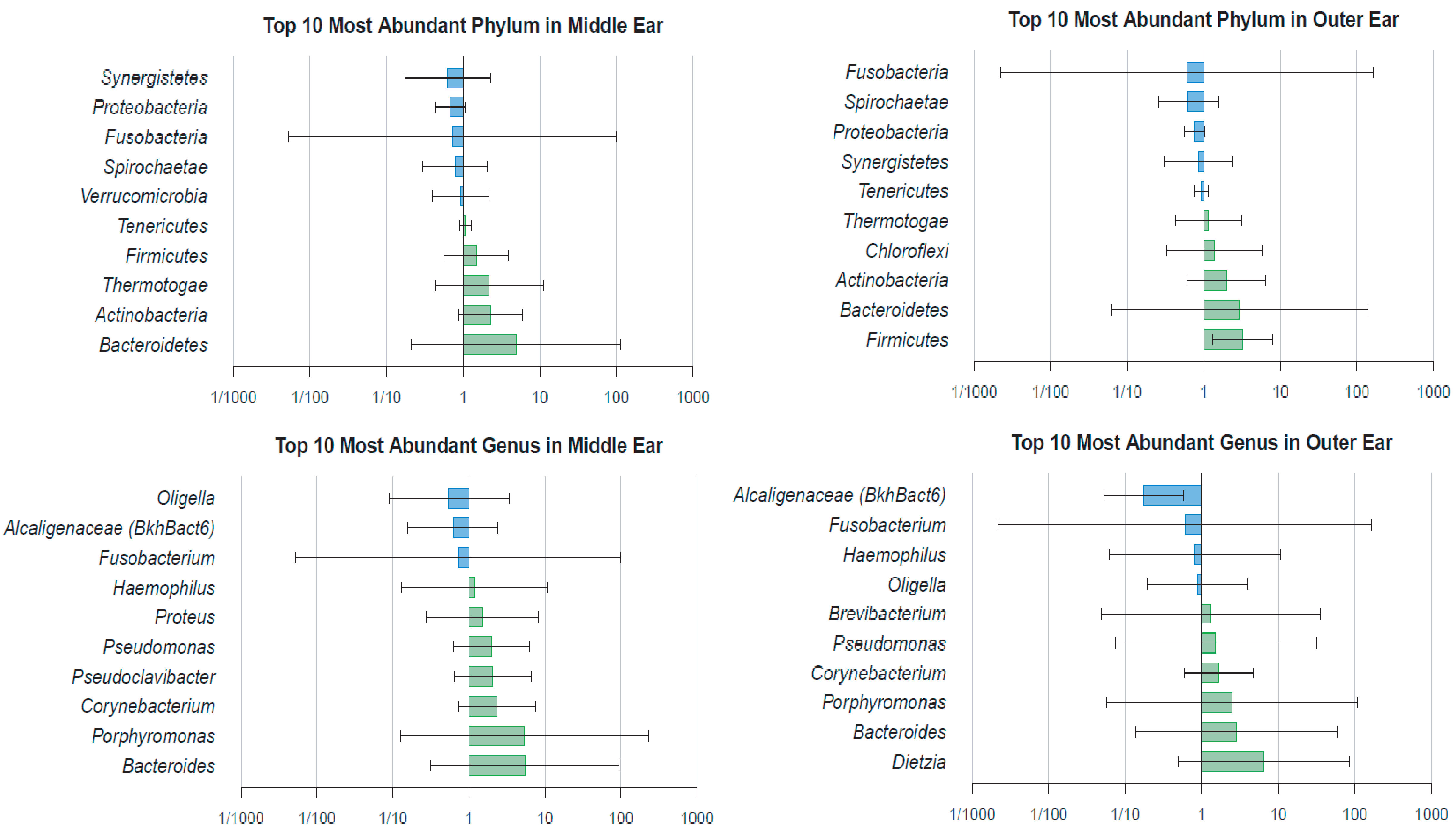

Fold Increase in Left Ear Samples Over Right Ear Samples
